# Seeded Growth Route to Noble Calcium Carbonate Nanocrystal

**DOI:** 10.1371/journal.pone.0144805

**Published:** 2015-12-23

**Authors:** Aminul Islam, Siow Hwa Teo, M. Aminur Rahman, Yun Hin Taufiq-Yap

**Affiliations:** 1 Catalysis and Science Research Center, Faculty of Science, University Putra Malaysia, 43400, UPM Serdang, Selangor, Malaysia; 2 Department of Chemistry, Faculty of Science, University Putra Malaysia, 43400, UPM Serdang, Selangor, Malaysia; 3 Laboratory of Marine Biotechnology, Institute of Bioscience, Universiti Putra Malaysia, 43400 UPM Serdang, Selangor, Malaysia; Institute for Materials Science, GERMANY

## Abstract

A solution-phase route has been considered as the most promising route to synthesize noble nanostructures. A majority of their synthesis approaches of calcium carbonate (CaCO_3_) are based on either using fungi or the CO_2_ bubbling methods. Here, we approached the preparation of nano-precipitated calcium carbonate single crystal from *salmacis sphaeroides* in the presence of zwitterionic or cationic biosurfactants without external source of CO_2_. The calcium carbonate crystals were rhombohedron structure and regularly shaped with side dimension ranging from 33–41 nm. The high degree of morphological control of CaCO_3_ nanocrystals suggested that surfactants are capable of strongly interacting with the CaCO_3_ surface and control the nucleation and growth direction of calcium carbonate nanocrystals. Finally, the mechanism of formation of nanocrystals in light of proposed routes was also discussed.

## Introduction

Over the past decades, nano-particles have received enormous attention due to its extensive technological applications [1]. Nano calcium carbonate synthesis have received significant concentration due to their industrial applications in the quantum dots, catalysis, flame retardant, clean-up of toxic contamination from chemical warfare, bone replacement and abundance of in nature [2–3]. Consequently, significant research on synthesizing nano-sized calcium carbonates at specific shape, morphologies, polymorphs has been performed. However, it is still a great challenge to obtain precisely engineer crystal shapes and properties tailored for a given application.

A considerable amount of research has been devoted to elucidate the nucleation and growth of nanoparticle in surfactant system [4]. It is now generally accepted that the single-crystal growth often occurs by the aggregation of precursor units rather than by ion-by ion growth [5–6]. Penn and Banfield in their discussion of TiO_2_ single crystal synthesis have emphasized the oriented attachment of nanoparticles. A similar observation has been made by other researchers [7–8] to synthesis single crystals of TiO_2_/SnO_2_and iron oxyhydroxide. However, the shape of crystals formed by the oriented attachment mechanism is very complicated and sensitive to the composition, temperature and pressure of the medium.

The interest in precipitation technique was dramatically quickened in the past decade to prepare nano CaCO_3_ from an aqueous suspension of calcium carbonate by bubbling pure CO_2_. Kim et al.[9] observed that metal carbonate [Ca^2+^]/[CO_3_^2−^] ratio and their saturation level controlled particle size and crystalline form of CaCO_3._ Song et al. [10] described the CaCO_3_ morphology change by varying the ionic atmosphere or the pH of the colloidal solution. As discussed above, laboratory processes have been emphasized mostly on the influence of external source of CO_2_ with desired metal-carbonate ratios to synthesis the CaCO_3_ nanoparticles. Therefore, searching for simpler solutions to synthesis CaCO_3_ nanocyrstals with controlled diameters is worthwhile.

An important advance in developing the synthesis strategy of nanoCaCO_3_from using fungi and actinomycetes as it produces reasonable amounts of CO_2_.A method has been used by Rautaray et al. [11] who attempted to use fungus as a source of carbonate (CO_3_^2-^) for the synthesis CaCO_3_ crystals. Other workers [12] have discussed similar phenomenon for the synthesis of nanoCaCO_3_ using *Thermomonospora sp*. stating that microorganisms reacts with the aqueous Ca^2+^ions to produce CaCO_3_nanocrystals. However, most preparation methods are tedious and complicated; do not easily produce monomorphological single crystals. Thus, the development of a technique to achieve this point of view is critically important. Thus, the focus is given in the present study to prepare nano calcium carbonate single crystal in the presence of surfactants from *salmacis sphaeroides* without external source of CO_2_ and the impact of surfactants on the crystal structure of CaCO_3._ The approaches the formation of nanocrystals in light of proposed routes was also adopted in this paper.

## Materials and Methods

### Materials

Waste *salmacis sphaeroides* shell was collected from Merambong shoal off, Tanjung Kupang (GPS Coordination: 01°34' N; 103°60' E), Johor, Malaysia having a mean body weight of 143.01±33.05 g (ranging from 90.55 to 200.35 g) and a mean test length of 72.85±5.08 mm (ranging between 63.88 and 80.24 mm). Dodecyl dimethyl betaine (BS-12) was bought from Sigma-Aldrich, Germany. CTAB were obtained from Marck, Germany. HPLC-grade deionized water used in all experiments. Analytical grade chemical reagents were used in this experiment. This work was conducted to the Catalysis Science and Technology Research Centre, Faculty of Science, University Putra Malaysia, Serdang, Selangor, Malaysia (GPS Coordination: 3°00'02.9"N 101°42'18.9"E). The vertebrate studies were not included in this work. We the authors confirmed that the field studies did not involve endangered or protected species and no specific permissions were required for these locations/activities. Merambong beach, Johor, Malaysia. It has been mentioned that this studies was not involved in human participants or tissue and vertebrate animals, embryos or tissues.

### Nanoparticle synthesis

**Fig 1:**
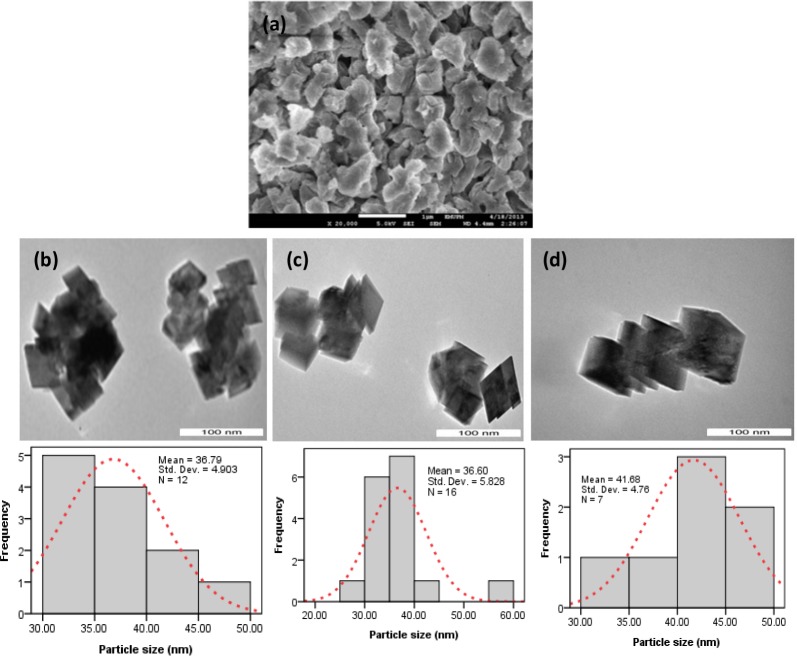
Starting material (a) and synthesized calcium carbonate nanocrystals: (b) B-1.5; (c) B-2.5; (d) B-3.5.

The preparation of CaCO_3_ nano-crystal was carried out in a conical flask using the reaction system *salmacis sphaeroides* shell-H_2_O-surfactants, as described in the literature [3,13]. The deionized water was used to clean *salmacis sphaeroides* shells and dried in an oven at 110°C for 2 h. The dried shells were then finely grounded by using a blender (Blendor-550, USA) and sieved to obtain micron-sized powders using 250 μm laboratory test sieve (Endecott Ltd., England).This micron sized particle was used as a starting material to synthesis nanopaticle (Fig 1A). First, 10 g of sieved powder with 100 mL water was stirred in a conical flask at 100 rpm for 30 min to form a colloidal solution. It should be mentioned that the pH of the aqueous solution of *salmacis sphaeroides* shell powder is ~9.3–9.5. The decomposition of *salmacis sphaeroides* was facilitated by adjusting pH ~6.5 with HCl of the colloidal solution, as reported previously [14]. 1mLof 1MHCl (1N) is added to change pH ~6.5. Then, both of the BS-12 and CTAB at different concentrations ranging from 1.5–3.5 (w/v%) was individually added into the colloidal solution and allowed to stir for 60 min. Islam et al. [13] in their discussion of the precipitation of CaCO_3_ have emphasized the electrostatic repulsion forces between the negative charge of carbonate ion and alkyl chain under basic condition (>pH = 10).Thus, the pH of the colloidal solution was increased to 10.5in this experiment to encourage precipitation of calcium carbonate in the colloidal solution, followed by stirring for 30 min to proceed precipitation of CaCO_3_. Subsequently, the slurry was filtered, washed and dried at 110°C in an oven for 12 h. The filtered sample were denoted as B-1.5, B-2.5, B-3.5 and C-1.5, C-2.5, C-3.5 where B indicates BS-12 assisted and C indicate CTAB assisted sample with different concentrations. Finally, the dried samples were calcined at 800°C for 4 h. The calcined samples were labelled as B_c_-1.5, B_c_-2.5, B_c_-3.5 and C_c_-1.5, C_c_-2.5, C_c_-3.5, where B_c_ indicates BS-12 assisted and C_c_ indicate CTAB assisted calcined sample with different concentrations.

### Characterizations

TEM images of the samples were obtained on a H7100- Hitachi operating at 200 kV. The samples for the TEM measurements were dropped and dried on a copper-coated grid. XRD measurements of the sample was carried out on XRD 6000, Shidmazu Corporation, Japan operated at a voltage of 30 kV and a current of 30 mA with Cu K_α_ radiation. X-ray fluorescence (XRF) spectrometer (Bruker, AXS) was used determine the chemical composition of the shell. To determine the absorption band, fourier transform infrared (FTIR) spectra of the powder sample was performed over the region 400–4000 cm^-1^ with a resolution of 4 cm^-1^ using Perkin Elmer (PC) Spectrum 100 FTIR spectrometer at room temperature.

Zeta potential of colloidal solution was determined by a 3000 HSA (Malvern) analyzer with light scattering at 90° angle. Thermogravimetric analysis (TGA) of the waste shell (*Salmacis sphaeroides*) powder was employed on a Mettler Toledo thermogravimetric analyser. The analysis was performed in a nitrogen atmosphere over a temperature range of 35–1000°C at a heating rate of 10°C min^-1^.

## Results and Discussion

A dispersity of calcium carbonate nanoparticle can be achieved by the presence of surfactant due to its amphiphilic structure adsorbed on the particles [2]. Surfactants have also been used as a capping agents to control the growth of colloidal particles [15]. Generally, the structure and properties of nanoparticles vary drastically depending on the concentration of surfactant. Increasing the surfactant concentration towards the point of neutrality increases the repulsive forces between the particles, and thus decreasing the size of particles, as reported by Pastoriza-Santo et al. [15]. By taking advantage of the property, two types of surfactants including CTAB (cationic) and (BS-12) zwitterionic surfactants were used in this study for the preparation of CaCO_3_ nanocrystal.

Fig 1A shows the starting material and calcium carbonate nanocrystals synthesized at different surfactant concentrations. The rhombohedral aragonite crystals assembled into a layered structure with a diameter of about 36–41 nm formed in the presence of a 1.5 wt % BS-12, as shown in Fig 1B. It has been reported by Cushing et al. ^1^ that the energy barrier formed at solid-liquid interface of the crystals causing the failure of crystals proximity in the colloidal solution, although Cases and Villieras [16] concluded from their works that the orientation of hydrophobic part of the surfactant molecules towards the solid surface and the projection of hydrophilic tail towards the aqueous environment causes loss of entropy which tend to repel crystal growth. Although similar size of crystals (33–39nm) were found in the sample with the addition of 2.5 and 3.5 wt.% BS-12, the crystal tends to be chain-like, as illustrated in Fig 1C and 1D. It is well known that the shape of calcium carbonate crystals could be influenced significantly by the polymer concentration.

**Fig 2:**
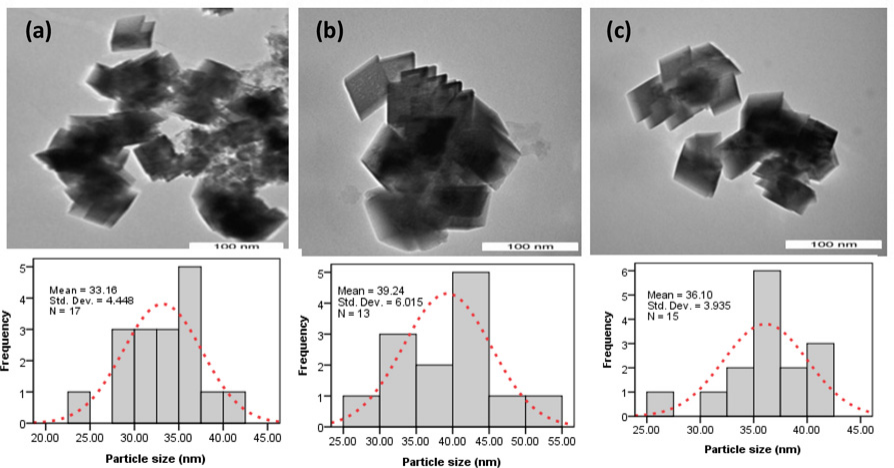
Typical calcium carbonate nanocrystals: (a) C-1.5;(b) C-2.5; (c) C-3.5.

The TEM results demonstrate that the crystals in the presence of CTAB (Fig 2A–2C) exhibit morphology similar to that presented in Fig 1B–1D with sizes of approximately 33–39 nm on all of the crystal faces. It is well known that the surfactant maintain necessary separation between precursor particles which prohibits the agglomeration of nanoparticles. A conclusion has been reached by Mishra et al. [17] that growth of nanoparticle affect greatly by the integrated effect of many factors including temperature, pressure, time and polymer concentration of colloidal particles. Mishra et al. [18] have suggested that grain boundaries of nanoparticle most likely offer the energetically favorable at heating condition to initiate the growth of nanoparticle although, it was concluded by the same research group [19] that the condition of the particle far from thermal equilibrium favor for nucleation of particle. May be mentioned that room temperature was used in this study to synthesize the nanoparticle. Thus, the uniform crystal morphology obtained in this study most likely could be due to the existence of thermal equilibrium conditions of the colloidal particle. An observation somewhat analogous to that reported in the case of metal oxide nanoparticle synthesis by other researchers [20–21].This high degree of morphological control demonstrates that surfactants are capable of strongly interacting with the CaCO_3_ surface and affecting nucleation and growth. Precipitation reaction is important to control the nucleation of particles. It has been shown by Gao and Goodman [22] that the surfactant adsorbed at the nanoparticle growth’s site inhibits the particle’s nucleation. Here, both the single crystalline structure and the smooth faces of crystals seem to suggest that the morphology could be associated with face selective surfactants adsorption to aragonite rhombohedral crystals at the initial stage of nucleation.

**Fig 3:**
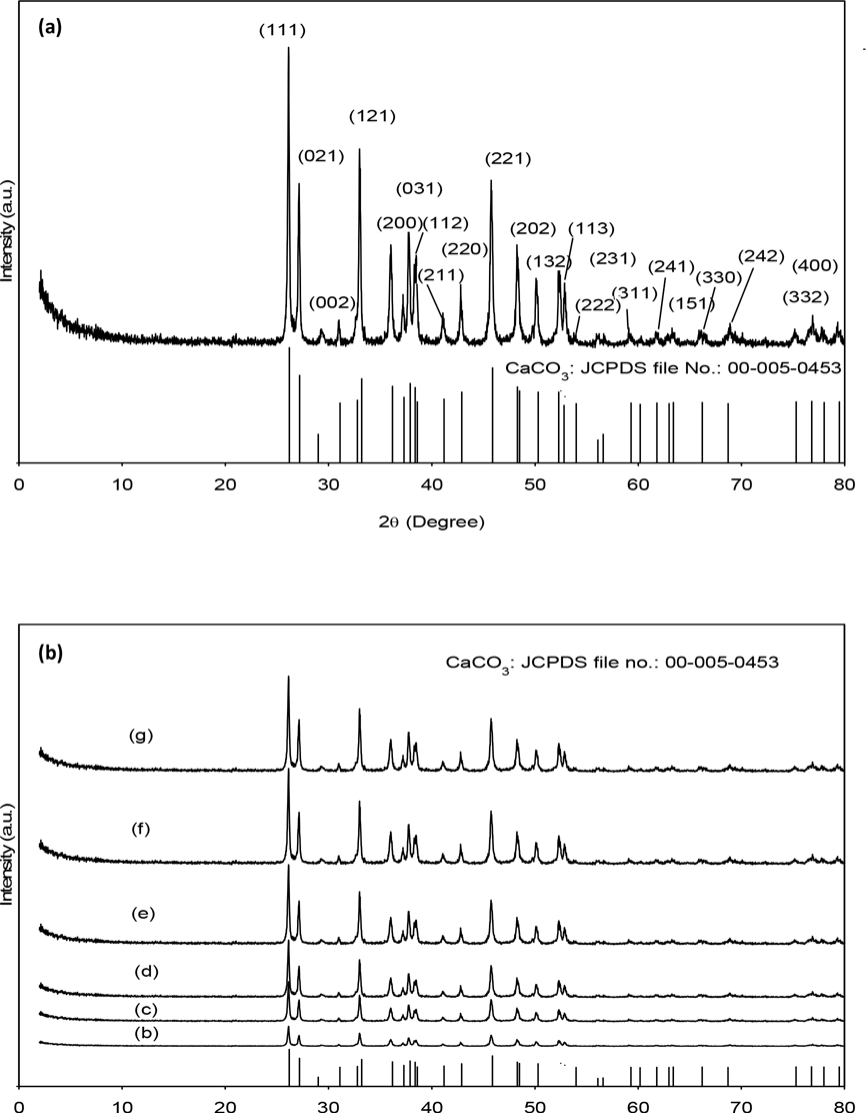
X-Ray diffraction pattern of (a) raw *salmacis sphaeroides* and treated shells: (b) B-1.5; (c) B-2.5; (d) B-3.5; (e) C-1.5;(f) C-2.5 and (g) C-3.5.

The crystal form of the CaCO_3_ is also determined in this study by conducting XRD analysis of the prepared nano powders (Fig 3). The XRD patterns of *salmacis sphaeroides*, as shown in Fig 3A exhibits the characteristic reflection of CaCO_3_ phase at 2*θ* values of 26.3°, 27.3°, 31.2°, 33.1°, 36.2°, 37.9°, 38.7°, 41.3°, 43.0°, 45.9°, 48.4°, 50.2°, 52.5°, 53.1°, 66.2° and 69.0° (JCPDS file: 00-005-0453).Although diffraction patterns of surfactant treated *salmacis sphaeroides* (Fig 3B–3G) were exhibited similar to Fig 3A,broader XRD peaks appeared in the pattern suggest decreasing crystallite size [23]. It is well known that polymer concentration may have influenced the growth and crystallization of particles [13,23].Chemical analysis using XRF has been conducted to estimate the mineral composition in *salmacis sphaeroides*. The finding demonstrates that the composition of *salmacis sphaeroides* mainly consists of Ca (99%). Small amounts of S, K and Sr was observed in the shells, as evident from XRF result. Hence, *salmacis sphaeroides* could be a potential natural carbonate-based materials to synthesis pure CaCO_3_ nanocrystals.

**Fig 4:**
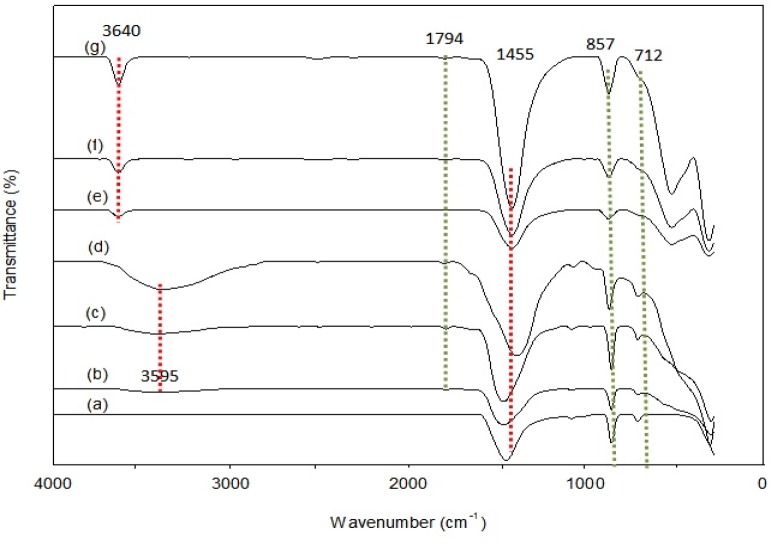
FT-IR spectra of (a) *raw salmacis sphaeroides* shell and treated shells (b) B-1.5; (c) B-2.5; (d) B-3.5; (e) C-1.5; (f) C-2.5 and (g) C-3.5.

To study the adsorption of surfactant on the surface of particle, FTIR was recorded and the spectra of *salmacis sphaeroides* powdered shell (a) and treated shells:(b) B-1.5; (c) B-2.5; (d) B-3.5; (e) C-1.5;(f) C-2.5 and (g) C-3.5are shown in Fig 4.The C–O stretching and bending modes of calcium carbonate appeared at 712 cm^− 1^, 857 cm^− 1^ and 1455 cm^− 1^[24]. Some additional vibrational modes of carbonate groups bond and metal ions observed at 1794 cm^-1^ [25].Though similar peaks were observed for untreated shells (Fig 4A) and BS-12 treated (Fig 4B–4D), the band at 3595 cm^-1^ in treated shell have been regarded as having alkyl groups at hydrophobic tail of surfactant attached to the particle during treatment. A similar result has been reported, stating that the carbonate ions react with the cations of the BS-12 and form dodecyldimethylaminoacetate carbonate [13].In the case of CTAB assisted sample (Fig 4E–4G), the peaks at 3640 cm^–1^ are ascribed to the long alkyl chain of CTAB (CH stretching region) and prove the presence of CTAB at the surface of calcium carbonate[3,24–25].No significant effect of surfactant on calcium carbonate polymorphism was observed, as evident from XRD and FT-IR studies.

To understand mechanism by which the presence of surfactants was investigated from the view of a measure of surface charges in the colloidal solution. ζ-potential values for the colloidal solution of *salmacis sphaeroides* particles at various concentrations of the two types of surfactants are shown in Table 1. The ζ-potential value for CaCO_3_ prepared at pH 9.5without the BS-12 and CTAB were 4 mV which was increased to 37 mV with decreasing pH to 6.5. The evidence for the presence of highly positively charged in the colloidal solution of CaCO_3_ has been observed Islam et al. [13] who concluded from their ζ-potential studies that the formation of high charge could be due to the dissociation of CaCO_3_ at pH 6.5. However, the addition of surfactants leads to a decrease in the ζ-potential value depending on the concentration of the surfactant used, as can be seen in Table 1. It is reasonable to state that the dodecyldimethylaminoacetate groups (HN—COO^-^) of BS-12 and cetyltrimethyl ammonium cations (NR_4_^+^Br^–^) of CTAB can bind the ionic phases of CaCO_3_ and thus, influence on the nucleation and growth of CaCO_3_ crystals. Other researchers [3,13] have discussed the similar phenomenon stating that CTAB dissociates into cetyltrimethyl ammonium cations which react with carbonate anion presence in the colloidal solution to precipitate calcium carbonate. FTIR measurements for the CTAB- and BS-12 modified calcium carbonate nanocrystals confirm that both BS-12 and CTAB are successfully adsorbed on the calcium carbonate surface. However, the amount of adsorbed charge in the case of BS-12 was higher than that of CTAB, as evident form Table 1. This could be explained by the ability of the dodecyldimethylaminoacetate groups of BS-12 to bind effectively to the Ca^2+^ ions on the calcium carbonate particle surface. Consequently, CTAB is less efficiently adsorbed on calcium carbonate particles.

**Table 1:** Zeta potential of the sample.

pH	Zeta potential without surfactant (mV)	Zeta potential with BS-12 (mV)			Zeta potential with CTAB (mV)		
	A-1.5	B-1.5	B-2.5	B-3.5	C-1.5	C-2.5	C-3.5
10.5	-4±1	-2	-3	-1	-3	-2	-2
9.5	-6±1	-2	-1	-1	-3	-2	-2
8.5	-11±2	-3	-2	-2	-5	-4	-3
7.5	-18±1	-3	-3	-1	-5	-4	-4
6.5	-37±1	4	3	2	5	5	4

B-1.5, B-2.5 and B-3.5 where B indicates BS-12 assisted Zeta potential value with the concentration (w/v%) of 1.5, 2.5 and 3.5, respectively.

C-1.5, C-2.5 and C-3.5 where C indicates CTAB assisted Zeta potential value with the concentration (w/v%) of 1.5, 2.5 and 3.5, respectively.

A-1.5 indicates the zeta potential value without surfactant at the concentration (w/v%) of 1.5.

By comparing the zeta potential, XRD, TEM and FTIR results, the mechanism of the nanocrystal formation was discussed. Several investigators [13,14] have shown that the decomposition of the CaCO_3_ facilitates at pH below 6.5 and carbonic acid split up into its constituents, releasing an excess of H^+^ to solution. Thus, the net effect of the dissolution of CO_2_ in solution is to increase concentrations of H^+^ resulting some CO_3_^2-^ react with H^+^ to become HCO_3_^–^ as follows (Eqs 1–5):

$${\text{CaCO}}_{3(\text{s})}+\;2{\text{H}}_{2}\text{O}\to {\text{Ca}(\text{OH})}_{2\;(\text{aq})}+\;{\text{H}}_{2}{\text{CO}}_{3\;(\text{aq})}$$ (1)

$${{\text{H}}_{2}\text{CO}}_{3\;(\text{aq})}↔{{{\text{HCO}}_{3}}^{-}}_{\left(\text{aq}\right)}+\;{{\text{H}}^{+}}_{\left(\text{aq}\right)}$$ (2)

$${{{\text{HCO}}_{3}}^{-}}_{\left(\text{aq}\right)}↔\;{{{\text{CO}}_{3}}^{2-}}_{\left(\text{aq}\right)}+{{\text{H}}^{+}}_{\left(\text{aq}\right)}$$ (3)

$${{\text{Ca}\left(\text{OH}\right)}_{2\;}}_{\left(\text{aq}\right)}↔\;{{\text{Ca}}^{2+}}_{\left(\text{aq}\right)}+{{2\text{OH}}^{-}}_{\left(\text{aq}\right)}$$ (4)

$${{{\text{CO}}_{3\;}}^{2-}}_{\left(\text{aq}\right)}+\;{{\text{H}}^{+}}_{\left(\text{aq}\right)}↔{{{\text{HCO}}_{3\;}}^{-}}_{\left(\text{aq}\right)}$$ (5)

It has been shown by several researchers [3,14] that the BS-12and CTAB in the suspensions at a basic condition dissociate into dodecyldimethylaminoacetate and cetyltrimethyl ammonium carbonate, respectively according to the following reaction equations (Eqs 6 and 7):

$$[{\text{CH}}_{3}{\left({\text{CH}}_{2}\right)}_{11}]{\text{N}}^{+}{\left({\text{CH}}_{3}\right)}_{2}{\text{CH}}_{2}{\text{COOH}}_{\left(\text{aq}\right)}+{\text{H}}_{2}\text{O}\to [{\text{CH}}_{3}{\left({\text{CH}}_{2}\right)}_{11}]\text{NH}{\left({\text{CH}}_{3}\right)}_{2}{\text{CH}}_{2}{{\text{COO}}^{-}}_{\left(\text{aq}\right)}+{\text{OH}}^{-}+{\text{H}}^{+}$$ (6)

$$[{\text{CH}}_{3}{\left({\text{CH}}_{2}\right)}_{15}]{\left({\text{CH}}_{3}\right)}_{3}\cdot {\text{HBr}}_{\left(\text{aq}\right)}+{\text{H}}_{2}\text{O}\to [{\text{CH}}_{3}{\left({\text{CH}}_{2}\right)}_{15}]{\left({\text{CH}}_{3}\right)}_{3}{{\text{N}}^{+}}_{\left(\text{aq}\right)}+\text{HBr}+\text{OH}$$ (7)

**Fig 5:**
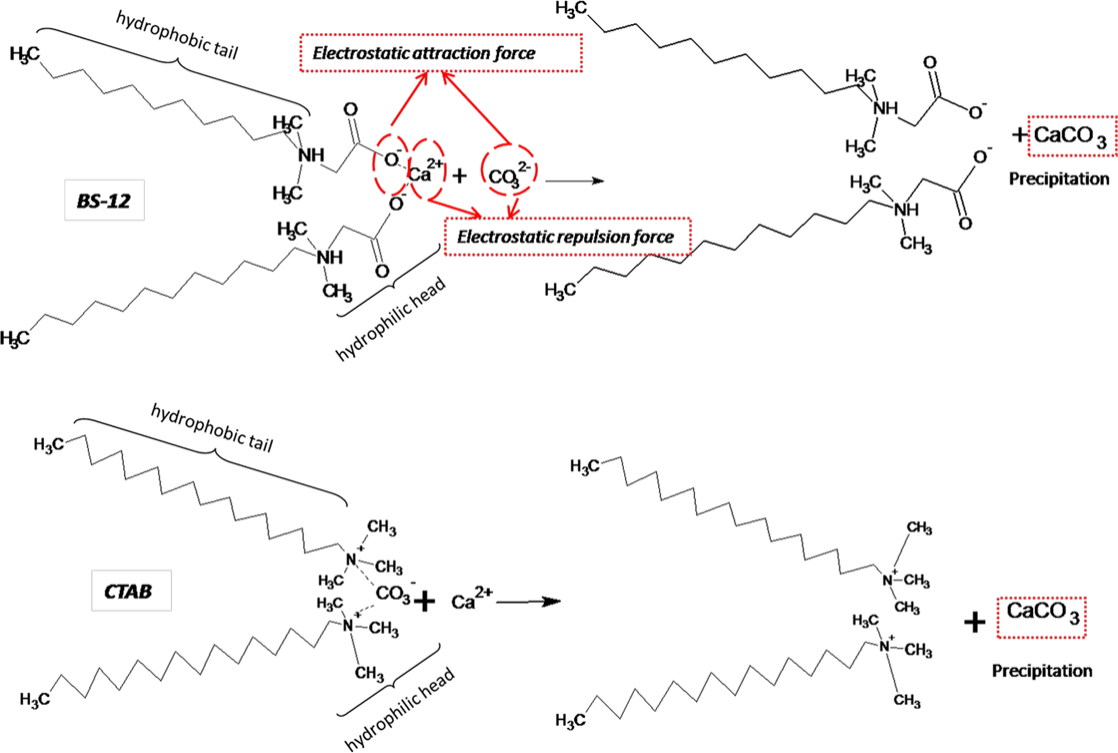
Mechanism for the precipitation of calcium carbonate.

El-Sheikh et al. [3] reported that the surface modifier concentration towards the point of surface neutrality provide an opportunity to precipitate the CaCO_3_ crystals. Islam et al. [13] in their discussion of the precipitation of CaCO_3_ have emphasized on the forces between the positive charged of carbonate ion and negative charged of hydrophilic head of alkyl chain; the present work suggest that facilitation of CaCO_3_ precipitation in the colloidal solution can be achieved at pH at ~10.5 as shown in Fig 5.

**Fig 6:**
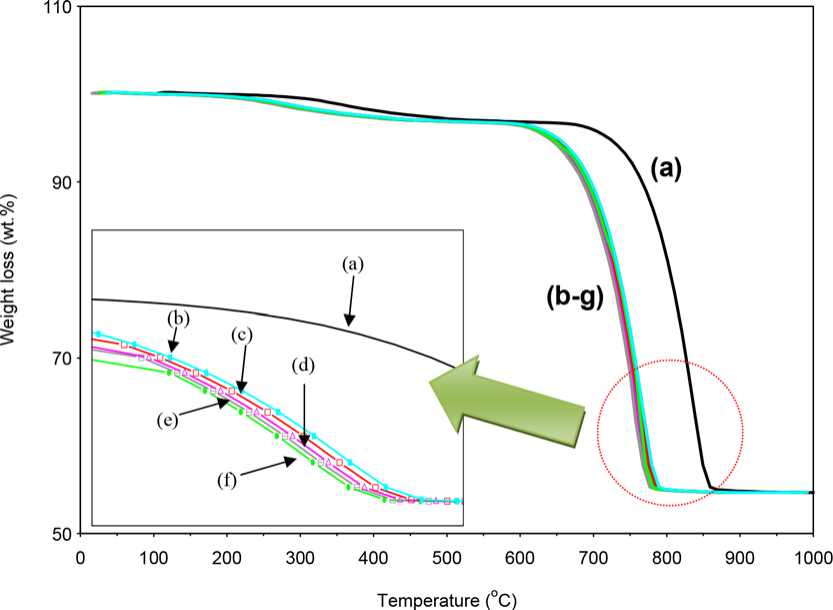
TGA of the *raw Salmacis sphaeroides* shell (a) and treated shells (b) B-1.5; (c) B-2.5; (d) B-3.5; (e) C-1.5;(f) C-2.5 and (g) C-3.5.

Further, the thermal dissociation of calcium carbonate nanocrystal into calcium oxide (CaO) has considerable technical importance due to its diverse applications, such as quantum dots, catalysis and field emission emitters [24–25].The crystal of calcium carbonate precipitated under different surfactant concentrations in this study transformed to CaO phase through calcination. Crystal-phase transformation of calcium carbonate is very well documented in the literature [26]. The calcium carbonate usually decomposes at temperatures over 800–850°C without fusion process as shown in Fig 6. The thermogravimetric curve TGA obtained for *Salmacis sphaeroides* (Fig 6A) showed a thermal stability up to 600°C with a small mass loss (1.8%) corresponding to volatile material. The thermal decomposition of calcium carbonate at temperature ranges 601 to 770°C with mass loss of 41.7% could be assigned to the decarbonization of calcium carbonate, representing by the following reaction (Eq 8).

$${{\text{CaCO}}_{3}}_{\left(\text{s}\right)}↔{\text{CaO}}_{\left(\text{s}\right)}+\;{{\text{CO}}_{2}}_{\left(\text{g}\right)}$$ (8)

However, the temperature corresponding to the weight loss of surfactant assistant samples as shown in Fig 6B–6G, were higher than that of *salmacis sphaeroides*, indicating smaller particle size. Previous studies found that the highest maximum decomposition rate of the CaCO_3_ particle could be introduced by the sample with smallest particle size. A similar conclusion has been reached by Islam et al. [13] who stated that the time required for the decomposition of smaller particles is shorter than the larger particles.

**Fig 7:**
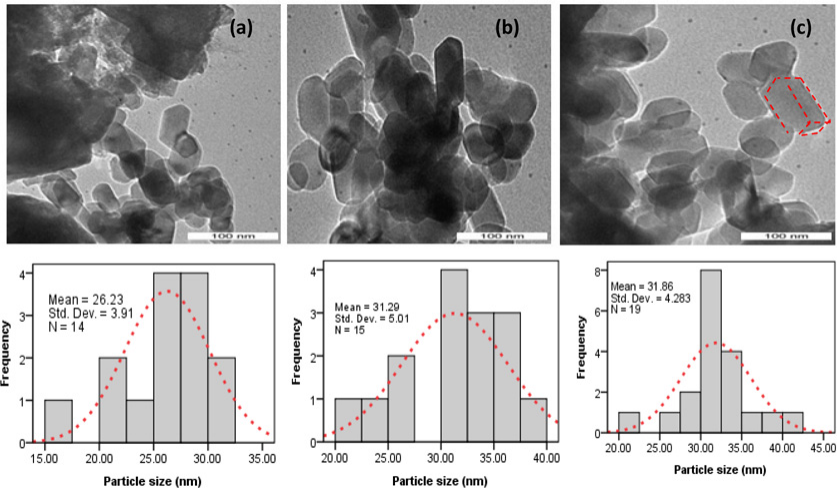
TEM of the calcined treated shells (a) B-1.5; (b) B-2.5; (c) B-3.5.

**Fig 8:**
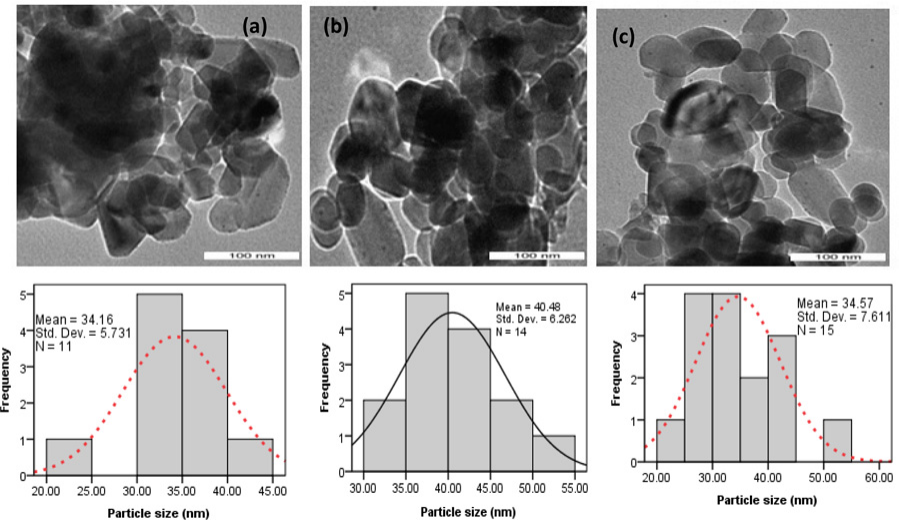
TEM of the calcined treated shells: (a) C-1.5; (b) C-2.5 and (c) C-3.5.

The TEM images of BS-12 assistant CaO sample (Fig 7A–7C) and CTAB assistant CaO sample (Fig 8A–8C) indicate that these nanoparticle are preferably crystalline. The image clearly revealed a noticeable transformation of shape from rhombohedral into monoclinic without their crystallinity after calcination at 800°C with the side dimension of 26–40 nm. It should also be noted that the size of the crystals becomes smaller after the calcination of CaCO_3_ nanocrystal. This observation indicates that the calcination temperature is important to the crystal size as well as shape of the CaCO_3_.Heavier rare earth elements (Yb, Lu) directly turns from rhabdophane to tetragonal at above 860°C [27]. In similar, hexagonal hydrated forms of BiPO_4_ transform into monazite-type compounds after heating [28].The transformation of CaCO_3_ is also confirmed in this study by conducting XRD and FTIR analyses of the prepared carbonate powders as shown in S1 Fig and S2 Fig.

## Conclusions

In summary, nano calcium carbonate with rhombohedric agaronite crystal have been synthesized from *salmacis sphaeroides* via seeded growth process. CTAB (cationic surfactant) and BS-12 (amphoteric surfactant) were used during the nanoparticle to modify size, morphology and dispersion stability of calcium carbonate nanocrystals. Formation mechanism of calcium carbonate nanocrystal suggests that multiple-amine head surfactants enables to precise control over the size and morphology of the nanocrystals without altering the chemical nature of the system. These nanocrystals exhibits rhombohedral aragonite crystal with sizes of approximately 33–41 nm on all of the crystal faces. Features including simplicity, low cost and versatility of the structures produced by this approach may open numerous areas not only in basic research but also for industrial application.

## Supporting Information

S1 Fig X-Ray diffraction pattern ofcalcined shells treated with surfactants: (a) B_c_-1.5; (b) B_c_-2.5; (c) B_c_-3.5; (d) C_c_-1.5;(e) Cc-2.5 and (f) C_c_-3.5. (TIF)

S2 Fig FT-IR spectra of calcined shells treated with surfactants: (a) B_c_-1.5; (b) B_c_-2.5; (c) B_c_-3.5; (d) C_c_-1.5;(e) Cc-2.5 and (f) C_c_-3.5. (TIF)
