# Supplementary figures and images for: Seeded Growth Route to Noble Calcium Carbonate Nanocrystal
Source: PLoS One. 2015 Dec 23;10(12):e0144805. doi: 10.1371/journal.pone.0144805 (PMC4689419; doi:10.1371/journal.pone.0144805)

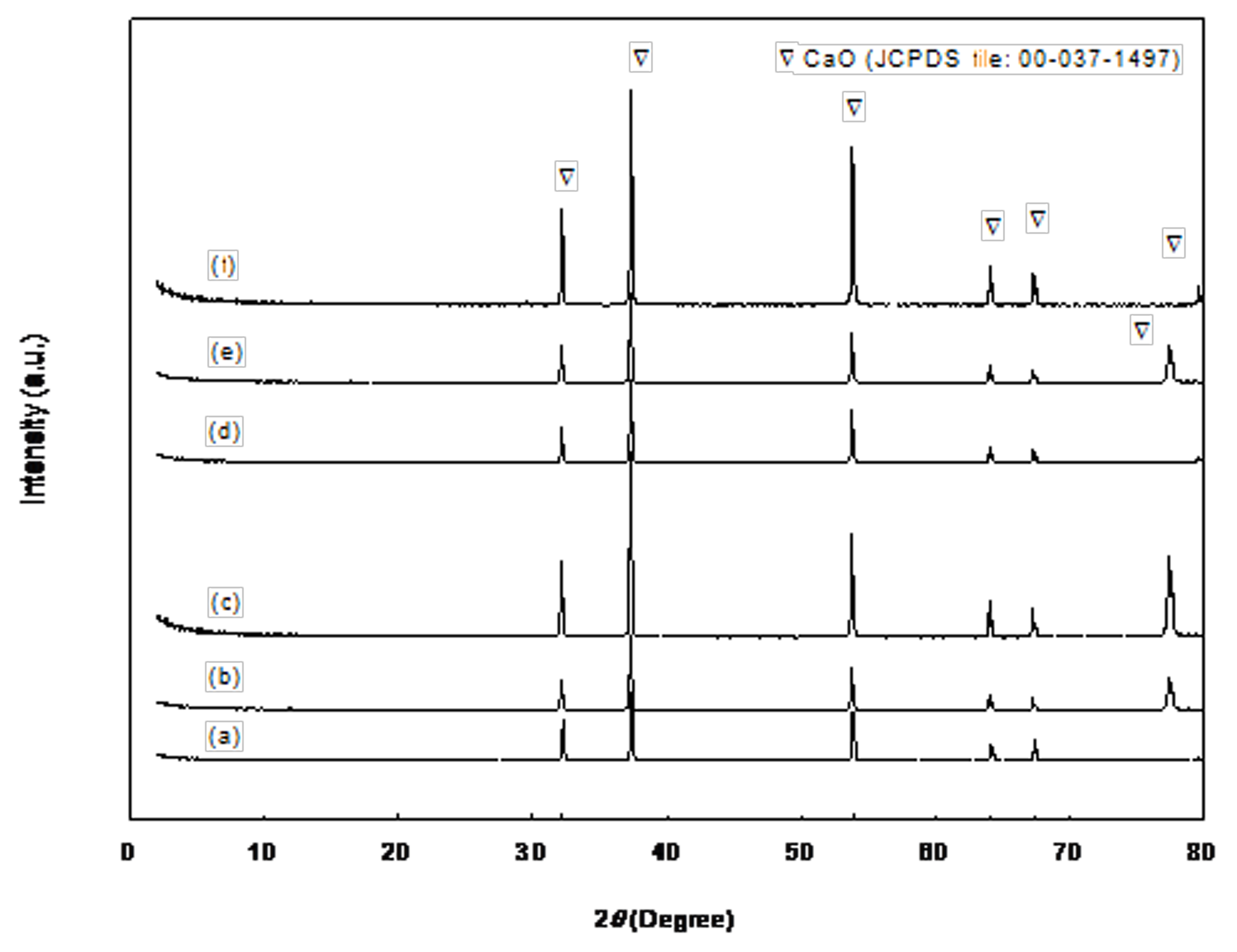

Supplement: S1 Fig — (TIF) [file pone.0144805.s001.tif]

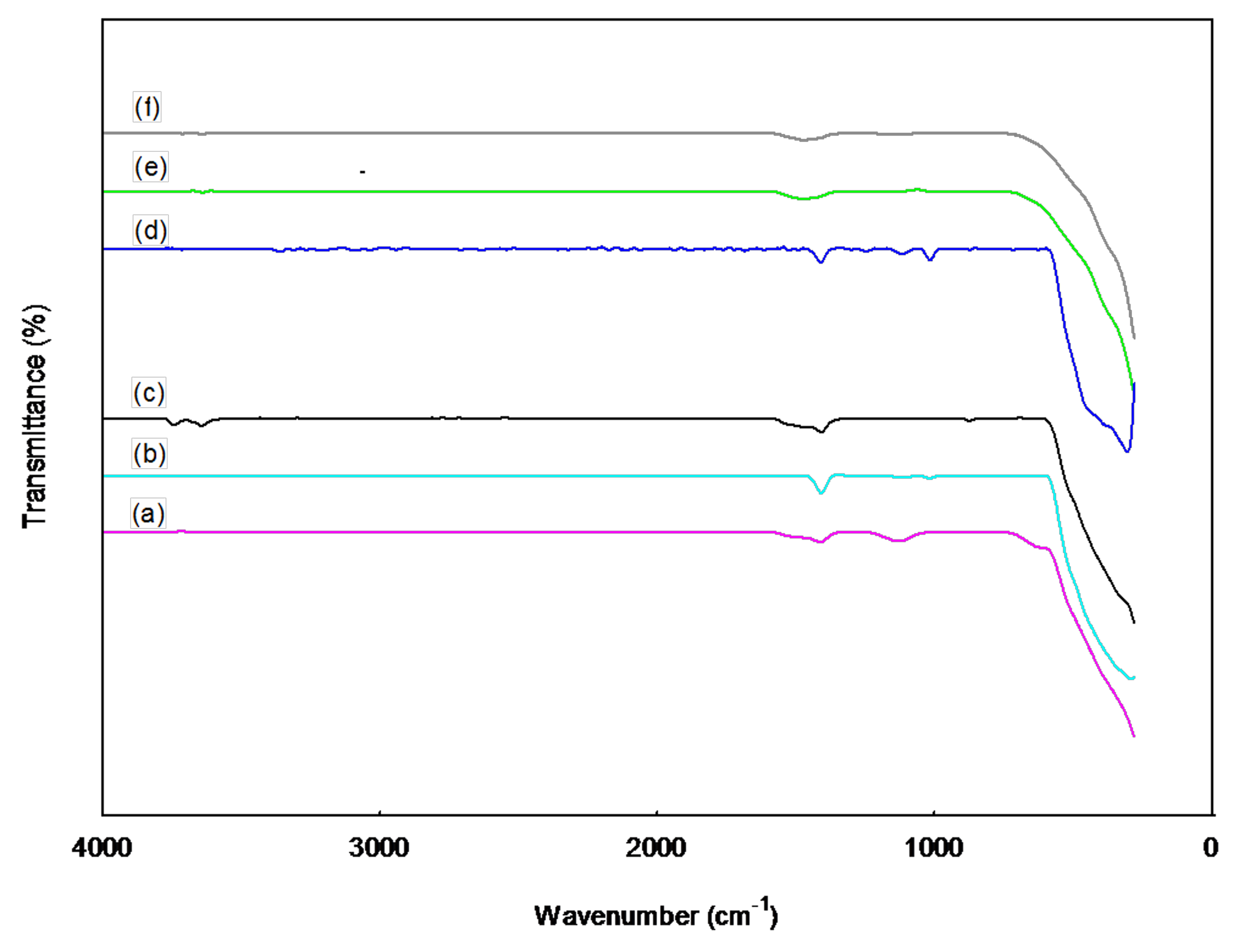

Supplement: S2 Fig — (TIF) [file pone.0144805.s002.tif]
